# Estimating Potential Incidence of MERS-CoV Associated with Hajj Pilgrims to Saudi Arabia, 2014

**DOI:** 10.1371/currents.outbreaks.c5c9c9abd636164a9b6fd4dbda974369

**Published:** 2014-11-24

**Authors:** Justin Lessler, Isabel Rodriguez-Barraquer, Derek A.T. Cummings, Tini Garske, Maria Van Kerkhove, Harriet Mills, Shaun Truelove, Rafat Hakeem, Ali Albarrak, Neil M. Ferguson, The MERS-CoV Scenario Modeling Working Group, Ricardo Aguas, Homud Algarni, Khalid AlHarbi, Simon Cauchemez, Hannah Clapham, Caitlin Collins, Anne Cori, Christl Donnelly, Christophe Fraser, Thibaut Jombart, Sean M. Moore, Pierre Nouvellet, Steven Riley, Henrik Salje, Abdulhafiz Turkistani

**Affiliations:** Johns Hopkins Bloomberg School of Public Health Baltimore, Maryland, USA; DepartJohns Hopkins Bloomberg School of Public Health Baltimore, Maryland, USA; Johns Hopkins Bloomberg School of Public Health Baltimore, Maryland, USA; MRC Centre for Outbreak Analysis and Modelling, Imperial College London, Faculty of Medicine, London, UK; MRC Centre for Outbreak Analysis and Modelling, Imperial College London, Faculty of Medicine, London, UK; MRC Centre for Outbreak Analysis and Modelling, Imperial College London, Faculty of Medicine, London, UK; Johns Hopkins Bloomberg School of Public Health Baltimore, Maryland, USA; Ministry of Health, Riyadh, Kingdom of Saudi Arabia; Ministry of Health, Riyadh, Kingdom of Saudi Arabia; MRC Centre for Outbreak Analysis and Modelling, Imperial College London, Faculty of Medicine, London, UK

**Keywords:** infectious disease, MERS-coronavirus

## Abstract

Between March and June 2014 the Kingdom of Saudi Arabia (KSA) had a large outbreak of MERS-CoV, renewing fears of a major outbreak during the Hajj this October. Using KSA Ministry of Health data, the MERS-CoV Scenario and Modeling Working Group forecast incidence under three scenarios. In the expected incidence scenario, we estimate 6.2 (95% Prediction Interval [PI]: 1–17) pilgrims will develop MERS-CoV symptoms during the Hajj, and 4.0 (95% PI: 0–12) foreign pilgrims will be infected but return home before developing symptoms. In the most pessimistic scenario, 47.6 (95% PI: 32–66) cases will develop symptoms during the Hajj, and 29.0 (95% PI: 17–43) will be infected but return home asymptomatic. Large numbers of MERS-CoV cases are unlikely to occur during the 2014 Hajj even under pessimistic assumptions, but careful monitoring is still needed to detect possible mass infection events and minimize introductions into other countries.

## Introduction

Appropriate response to novel or emerging infectious diseases is one of the most difficult challenges faced by public health organizations. Due to limited data, mathematical models are often used to make projections. However, these models also rely on data, and are only as good as our understanding of the disease process. Careful synthesis of the available information, combined with development of planning scenarios provide a pathway to making useful forecasts in the face of significant public health threats. This manuscript reports on one such synthesis, an analysis performed in support of the Kingdom of Saudi Arabia Ministry of Health in the lead up to the 2014 Hajj. Due to the different time lines of public health response and publication, this analysis is appearing after the 2014 Hajj, and we discuss what actually happened in the conclusion. The main analysis is presented as it was performed to give an unbiased assessment of how the analysis was approached.

To date, more than 800 laboratory-confirmed cases of MERS-CoV have been reported to the World Health Organization, with over 80% being reported by the Kingdom of Saudi Arabia (KSA) [http://www.who.int/csr/disease/coronavirus_infections/]. The mortality rate in reported infections has been high: as of July 25, 2014, 41% of the 721 cases detected in KSA have died.

The spring of 2014 saw a large increase in MERS-CoV incidence in KSA associated with multiple outbreaks across the country, including large outbreaks in Jeddah and Riyadh. A total of 525 cases were confirmed between March 1 and June 30, 2014, over twice the number previously identified (Figure 1). After intensive efforts to raise awareness, enhance surveillance and improve compliance with hospital infection control procedures, these outbreaks appear to have been brought under control.

**MERS-CoV cases reported by the KSA Ministry of Health between June 1, 2012 and June 30, 2014.**
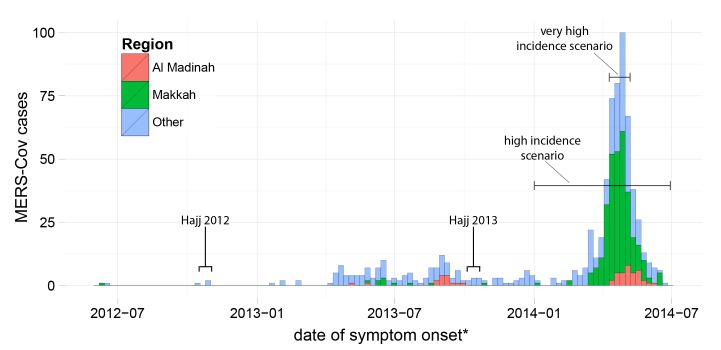
Horizontal lines indicate time periods over which incidence in the Makkah region (Jeddah city included ) was averaged to obtain incidence rates for the high and very high incidence scenarios. * - asymptomatic cases and cases with an otherwise missing date of onset are shown at the date the case first tested positive for MERS-CoV.

The scale of the 2014 outbreak has renewed concerns about the potential for MERS-CoV transmission in pilgrims visiting KSA for the Hajj in October 2014. There are reasons to be concerned. Two to three million Saudis and foreign pilgrims will visit Makkah and Madinah, congregating in densities of up to seven per square meter during religious events.^1^ Most pilgrims are older males, many with underlying conditions,^2^ common features of severe MERS-CoV cases. There is evidence of increased transmission efficiency of respiratory infections among Hajj pilgrims.^3^ A significant proportion of pilgrims seek or require health care while in KSA (Figure 2), and health care associated transmission has helped to drive previous outbreaks.^4^ Finally, pilgrims infected while in KSA may return to their home countries while still incubating the infection, potentially seeding outbreaks.

Given the risks, it may seem surprising that in 2012 and 2013 the Hajj was not associated with large increases in MERS-CoV incidence (Figure 1) or significant numbers of cases among foreign pilgrims.^5^^,^ 6 ^,^ 7 There are several possible explanations: incidence in KSA was low at the beginning of the Hajj in both years (Figure 1); pilgrims do not stay in KSA for the Hajj long enough for multiple generations of transmission to occur; and the movement of pilgrims is tightly controlled, limiting contact with possible zoonotic or environmental sources and community cases. However, the sheer number and density of pilgrims and the potential for global dispersal of the virus to previously unaffected countries means the risks need to be rigorously reassessed and response plans formulated. Thus, the Saudi government conducted intensive surveillance for MERS-CoV at health care centers during the Hajj and has developed response plans for handling any detected cases.

**Healthcare and the Hajj:**
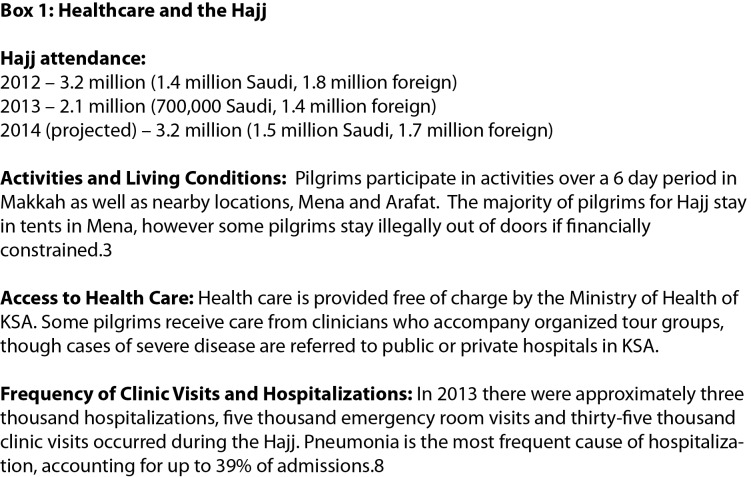

Here we present projections developed as part of real-time modeling and epidemiological analysis performed in support of the KSA Ministry of Health’s MERS-CoV planning efforts. We assess the risk of MERS-CoV incidence in KSA during the Hajj, the incidence among pilgrims returning to their home countries, and the testing burden associated with MERS-CoV surveillance during the Hajj. Original projections were performed independently by researchers at Imperial College and the Johns Hopkins Bloomberg School of Public Health and further validated by comparison with MERS-CoV incidence among foreign Umrah pilgrims visiting KSA during preceding Ramadan (which occurred July 2014 this year). The estimates presented here represent a synthesis of the work of these two groups. This paper aims to both help public health organizations plan for potential risks associates with the Hajj, and illustrate how thoughtful use of limited data can be used to plan the response to emerging infectious diseases.

## Methods

Estimated MERS-CoV Incidence During the Hajj 

2014Incidence rates and epidemic curves are based on a complete line list of all individuals with confirmed MERS-CoV infection in KSA between June 7, 2012 and June 30, 2014 provided by the KSA MoH (Figure 1). Age-specific incidence rates of MERS-CoV infection were calculated for each region of KSA. A parallel data set of all confirmed MERS-CoV infections among foreign Umrah pilgrims reported by the WHO or in ProMED between March 1 and June 30, 2014 was assembled by two independent reviewers. KSA residents were excluded due to difficulties in distinguishing infection associated with performing Umrah from that associated with other activities. Data on the number and age distribution of Umrah pilgrims was provided by the KSA MoH.

These data sets were used to project MERS-CoV incidence in Hajj pilgrims under three scenarios: (1) *Expected/Low Incidence*: the weekly risk of infection will match that seen among foreign Umrah pilgrims between June 1 and August 28, 2014; (2) *High Incidence*: the weekly risk of infection will match the age-adjusted average experienced by residents of the Makkah region) between January 1 and June 30, 2014 (figure 3); (3) *Very High Incidence*: the weekly risk of infection will match the age-adjusted average for the Makkah region during the four peak incidence weeks of 2014 (April 10 to May 7, 2014). Non-Saudis are assumed to have no risk of MERS-CoV infection prior to the Hajj, and Saudis are assumed either to have the risk seen in the Makkah region during June 2014 (*Expected/Low *scenario) or the same risk as during their Hajj visit (*High* and *Very High* scenarios). We report the expected number of pilgrims developing symptoms during the Hajj under each scenario, including KSA citizens infected before attending.

**Illustration of transformation of age specific MERS-CoV incidence in the general population of the Makkah region to incidence in foreign pilgrims to the Hajj.**
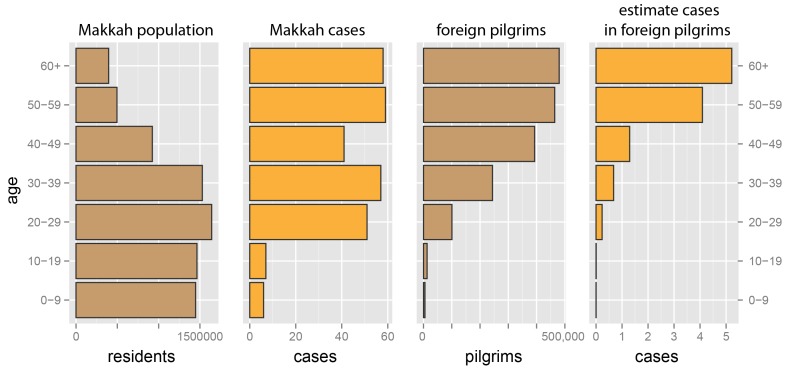
Here we show the population of Makkah, MERS cases occurring in the first 27 weeks of 2014, expected number of foreign pilgrims, and expected number of cases in foreign pilgrims. Hajj pilgrims are at risk for two weeks; hence, for each age category, C, the expected incidence in Hajj pilgrims (without onward transmission) is (cases in C)/(population in C × 27 weeks) × (foreign pilgrims in C × 2 weeks).

Based on the incubation period of MERS-CoV and the distribution of Hajj attendees by country reported for 2008,^9^^,^ 10 we project the number of foreign pilgrims infected during the Hajj developing symptoms before and after returning to their home country. The incubation period of MERS-CoV is assumed to follow a lognormal distribution with a mean of 5.6 days and dispersion of 1.63.^9^Based on planning scenarios provided by the KSA MoH, we assume an expected 3.2 million Hajj attendees (1.5 million Saudis and 1.7 million foreigners), that foreigners stay for an average of 14 days, and that Saudis stay for an average of 7 days. On average, 500,000 foreign visitors are assumed to attend Umrah each month, staying for an average of one week except in Ramadan where it may extend to two weeks. Background infection rates are considered to be constant throughout a pilgrim’s entire stay in KSA. We account for the possibility of onward transmission during the Hajj by projecting additional generations of transmission among pilgrims. We assume an average reproductive number of 0.63 and mean generation time of 10-11 days,^11^ and onward transmission is assumed to be truncated when pilgrims depart KSA.

Projections and 95% prediction intervals were created through stochastic simulations (see supplemental material).

Health facility use and severe respiratory disease during the Hajj

Two teams performed independent reviews of the literature to identify papers reporting incidence of pneumonia and other respiratory syndromes during the Hajj. Papers were reviewed for reports of proportions of hospitalized pilgrims with pneumonia or acute respiratory infection (ARI) and the proportion of those seeking outpatient or emergency care with pneumonia or influenza like illness (ILI). No reports of ILI rates specifically in hospitalized patients were identified. When outpatient data was unavailable, the rate among clinic and emergency room attendees was assumed to be that recorded in the overall pilgrim population. To calculate the expected number of potential suspect MERS-CoV cases, these rates were applied to the number of pilgrims seen as inpatients (i.e., hospitalized) and outpatients (i.e., visiting the emergency room or an outpatient clinic) during the 2013 Hajj.

Supporting Data

Data on MERS-CoV incidence in KSA, expected Hajj and Umrah attendance patterns and patterns of health care utilization during the Hajj were provided directly by the KSA Ministry of Health as part of an ongoing collaboration, unless otherwise noted.

## Results

For each scenario, baseline risk of infection (not including onward transmission) and supporting data is shown in Table 1. Though age specific risks are assumed to be the same in KSA resident and foreign pilgrims, infection risk per one-million attendees is projected to be higher among foreign pilgrims due to their generally older age (Figure 3). Under the expected incidence scenario, we predict that 6.2 pilgrims (95% prediction interval [PI], 1-17) will develop MERS-CoV during the 2014 Hajj (Table 2). Under the high incidence scenario, we would expect to see 11.7 cases (95% PI, 5-20); and 47.6 cases (95% PI, 32-66) under the very high incidence scenario. It is expected that 45% of foreign pilgrims infected during the Hajj will not develop symptoms until returning to their home country (Table 2).

**Table 1: Summary of the three scenarios used to project incidence of MERS-CoV among pilgrims during the Hajj.** 

**** **Scenario**	**Observed** **Population**	**Observed period**	**Number of cases**	**Infection risk in KSA pilgrims per 1,000,000 person weeks**	**Infection risk in foreign pilgrims per 1,000,000 person weeks**
**1. Expected - Low**	foreign Umrah pilgrims	March 1 – June 30, 2014	9	0.85	2.1
**2. High Incidence**	KSA residents	January 1 – June 30, 2014	280^*^	1.9	3.7
**3. Very high Incidence**	KSA residents	April 10 – May 7, 2014	182	7.6	14.9
* - analysis based on the 279 pilgrims whose age was recorded in the case listing.					

**Table 2: Projections of clinical incidence among Saudi and foreign Hajj pilgrims.** Specific incidence is shown for the six countries that account for the majority of Hajj attendees. Ninety-five percent prediction intervals based on 10,000 simulations accounting for parameter uncertainty and stochastic variability in infection and the number and demographics of Hajj attendees (see supplemental methods).

	**Expected/Low** **Incidence**		**High** **Incidence**		**Very High** **Incidence**	
	**Hajj pilgrims developing symptoms in KSA** ****	**Hajj pilgrims developing symptoms after returning** ****	**Hajj pilgrims developing symptoms in KSA** ****	**Hajj pilgrims developing symptoms after returning** ****	**Hajj pilgrims developing symptoms in KSA** ****	**Hajj pilgrims developing symptoms after returning** ****
**Total**	6.2(1, 17)	5.2(0, 14)	11.7(5, 20)	9.5(4, 17)	47.6(32, 66)	40.0(26, 56)
KSA Residents^*^	1.2(0, 3)	1.2(0, 4)	2.9(0, 7)	2.4(0, 7)	11.8(6, 19)	11.0(4, 20)
Visitors	5(0, 15)	4(0,12)	8.7(3, 16)	7.1(2, 14)	35.7(22, 52)	29.0(17, 43)
*Indonesia*	0.6(0,3)	0.5(0 ,2)	1.1(0, 4)	0.9(0, 3)	4.5(1, 9)	3.6(0, 8)
*India*	0.5(0, 2)	0.4(0, 2)	0.9(0, 3)	0.7(0, 3)	3.6(0, 8)	2.9(0, 7)
*Pakistan*	0.5(0,3)	0.4(0, 2)	0.9(0, 3)	0.7(0, 3)	3.5(0, 8)	2.8(0, 7)
*Turkey*	0.4(0, 2)	0.3(0, 2)	0.7(0, 3)	0.6(0, 2)	2.8(0, 7)	2.2(0, 6)
*Iran*	0.3(0, 2)	0.2(0, 2)	0.6(0,2)	0.4(0, 2)	2.3(0, 6)	1.8(0, 5)
*Nigeria*	0.3(0, 2)	0.2(0, 2)	0.5(0, 2)	0.4(0, 2)	2.0(0, 5)	1.6(0, 5)
*Other*	2.4(0,8)	1.9(0, 7)	4.2(1, 9)	3.4(0, 8)	17.1(9, 27)	14.0(7, 23)
* - KSA residents developing symptoms during the Hajj include pilgrims infected prior to attending the Hajj. **						

Even if no cases of MERS-CoV occur during the Hajj, a significant percentage of patients will need to be tested for MERS-CoV based on clinical symptoms (Table 3). Inpatients and outpatients with pneumonia and influenza-like illness due to other causes could all potentially meet the criteria for MERS-CoV testing. In 2013 there were 3,116 hospitalizations, 4,799 emergency room visits and 34,550 clinic visits recorded by the KSA Ministry of Health during the Hajj. If rates are similar during the 2014 Hajj, and all pneumonia and ILI cases are tested for MERS-CoV we expect 511 to 1,402 inpatients and 1,919 to 8,209 outpatients will be tested (based on high and low percentages shown in Table 3, accounting for Poisson variation).

**Table 3: Percentage of inpatients and outpatients potentially tested for MERS-CoV due to another underlying condition.** Mean estimates are the mean of all rates reported in the literature. High and low estimates are based on the low and high reported incidence rates.

	**Inpatients ** **(pneumonia | ARI)**	**Outpatients** **(pneumonia | ILI)**
**Mean Estimate**	27.8%(24.6% | 3.2%)	11.1%(0.4% | 10.7%)
**High Estimate**	42.7%(39.4% | 3.3%)	20.4%(0.8% | 19.6%)
**Low Estimate**	17.9%(14.8% | 3.1%)	5.1%(0.2% | 4.9%)

## Discussion

The large number and high density of visitors associated with the Hajj has led to understandable concerns about the potential for an explosive outbreak of MERS-CoV. Quantitative analysis is useful to assess the potential scale of risk associated with events such as the Hajj. At first glance, it may seem surprising that we project less than 100 individuals will develop MERS-CoV during the 2014 Hajj, even in our most pessimistic scenarios. This value is relatively low in part due to the short duration of visit of pilgrims to the Hajj (7-14 days), which limits the opportunities for secondary transmission (Figure 4). Thus we expect that most MERS-CoV cases occurring in pilgrims will arise due to exposure to cases or infected other source already prevalent in KSA. While the Spring of 2014 saw large hospital-centered outbreaks of the virus across KSA, improvements in diagnosis and compliance with infection control practice were associated with a decline in incidence to near zero by the end of July. If this trend continues, the infection risk to KSA residents and visitors over the next few months can be expected to be very low (perhaps even lower than the lowest scenario considered). Furthermore, the well-developed health care and surveillance systems put in place for Hajj and the regulations on visitor movements may result in exposure risks for pilgrims being rather lower than for the general KSA population - which may explain in part why no cases of MERS-CoV occurred during the Hajj during 2012 or 2013.

Policymakers in KSA need to plan for the reasonable worst-case scenario. Therefore, we made highly pessimistic assumptions to generate our highest incidence estimates of Hajj-associated MERS-CoV cases: (a) Hajj pilgrims will face the same daily risk of infection experienced by residents in the worst affected Makkah region of KSA during the highest incidence 4 week period of 2014; (b) despite the improvements in infection prevention and control implemented this year, secondary transmission will occur at the same levels seen in 2012 and 2013; (c) that age-specific attack rates in pilgrims will mirror those seen in the KSA population over the last 2 years. Thus while the resulting projections (of under 100 cases) are appropriate for planning purposes, they are not likely to be realized. Our best estimate is that around six pilgrims (95% PI, 1-17) will develop symptoms of MERS-CoV during the 2014 Hajj, based on the numbers of cases reported in foreign pilgrims visiting KSA for Umrah between June and August 2014. However, even this figure may be pessimistic, given recent reductions in MERS-CoV incidence in KSA and among visitors (including Umrah pilgrims).

Questions remain about the epidemiology of MERS-CoV. Transmission between humans has driven outbreaks in healthcare settings,^9^^,^ 12 but the role of human-to-human transmission and zoonotic infections in community acquired cases remains unclear. There is increasing evidence that camels play an important role,^13^^,^ 14 but many cases have no known animal exposure and person-to-person transmission has occurred outside of hospitals.^15^ Hence, our understanding and ability to quantify the relative contribution of these different routes transmission is limited, and we have even less understanding of how the special circumstances of the Hajj will affect the risk of infection. Given these uncertainties, we adopted a relatively simple, but empirically driven, approach to risk assessment in this work; relying on careful synthesis of the available data and important planning scenarios rather than sophisticated mathematical models. It is possible that some of the model assumptions in this simplified approach underestimate the risk of unexpectedly larger transmission events, as have been seen in some hospitals. To account for this possibility we performed a sensitivity analysis where we repeated the model using a highly over-dispersed distribution for onward transmission; the results of this analysis were not qualitatively different from the main analysis (see Appendix 2).

While MERS-CoV incidence is likely to be low, it is expected that thousands of pilgrims will be tested for the disease. Though nearly all tests will be negative;^6^ this testing will be critical as MERS-CoV has proven its ability to transmit efficiently in healthcare settings if appropriate infection control procedures are not followed or not in place. Hence, cases must be identified rapidly and appropriate contact precautions put in place as quickly as possible. Furthermore, despite our projections of low incidence, the high density of pilgrims might offer the potential for super-spreading events. Though not seen thus far for MERS-CoV, such events can be highly challenging to contain. Careful surveillance and testing will minimize the number of MERS-CoV infected individuals attending mass gatherings, and quickly detect any surge in transmission. Virological surveillance is also critical and rapid sequencing of viral isolates will provide timely information on whether any apparent changes in the epidemiology of MERS-CoV, such as increased transmissibility, are associated with significant evolutionary changes in the virus.

Ultimately, no Hajj associated cases were detected in Saudi Arabia during 2014, and while three suspect cases occurred among foreign attendees returning home, none was confirmed. This is in line with our expected/low incidence scenario. In retrospect, some might argue that preparing for more cases was a waste of resources. However, had an outbreak occurred and KSA not been prepared they would be subject to severe criticism and precipitated a global public health crisis, similar to what resulted from the early response to Ebola in West Africa. The type of scenario planning reported in this manuscript is essential to any public health response, and while we can always hope that our most optimistic forecasts are realized, we should never assume this is the case and work hard to develop data grounded scenarios that capture the worst reasonable outcome given what we know about the disease threats. There is no indication that the MERS-CoV threat will have subsided by the next Hajj, and there have been an increasing number of case reports through October, raising concerns as we approach the apparent high season for MERS-COV incidence in spring 2015. It is essential that we continue to update and consult planning scenarios, so that we are prepared to confront and contain the disease whatever may come.

**Is a major outbreak of MERS-CoV likely during the Hajj?:**
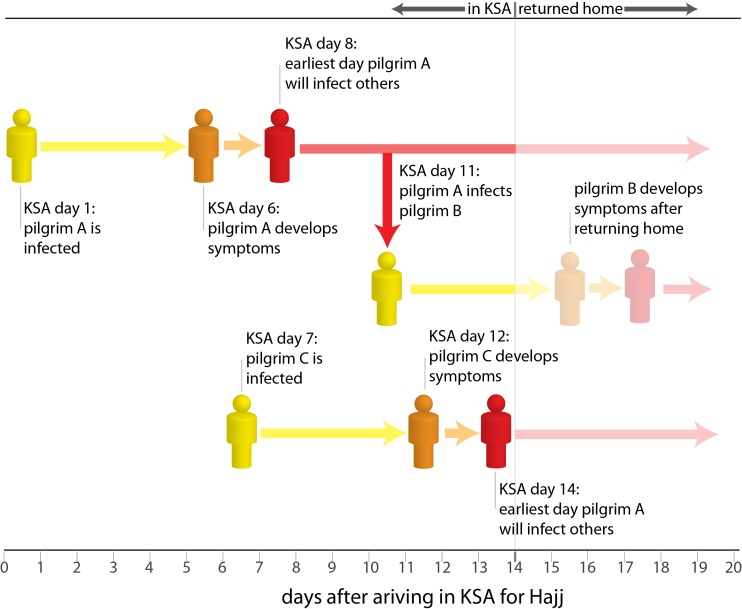
**Factors making a major outbreak unlikely:** The generation time of an infectious disease is the time between when an individual is infected and the time at which they infect any subsequent cases. The average generation time for MERS-CoV is estimated to be around 10 days, and it is highly unlikely that a case will cause any subsequent infections prior to 7 days after infection.11 Only Hajj pilgrims infected early in their stay in KSA will have the opportunity to infect additional pilgrims themselves, and those they infect are extremely unlikely to cause additional infections. Hence, it is unlikely to see more than two generations of transmission during the Hajj, an insufficient number of generations to cause a major outbreak. Most Hajj pilgrims do not mix with locals or visit locations other than the Hajj area (Makkah and Madinah), reducing their opportunities to be infected. The figure provides an example: pilgrim A is infected on the first day of his stay in KSA for the Hajj, but is unlikely to infect anyone else until 7 days later, 8 days after arriving in KSA. Pilgrim A eventually infects pilgrim B 10 days after being infected (the average generation time of MERS-CoV). Pilgrim B does not develop symptoms until returning home from the Hajj, and causes no onward transmission during his stay in KSA. Pilgrim C is only infected on day 7 of her visit, but does not cause secondary infections at the Hajj as she only develops symptoms just before leaving KSA. There is the possibility of onward transmission, and potentially large outbreaks, after pilgrims return to their home countries; however, such outbreaks lie outside the scope of this analysis.** Factors that could lead to a major outbreak:** While multiple generations of transmission are unlikely, some types of unexpected events could lead to significantly more cases than predicted here. Super spreading events where a single MERS-CoV infected animal or human causes a large number of cases are possible. Viral mutations that shorten the generation time or lead to more efficient transmission are unlikely, but could also cause a significant outbreak if they were to occur.

## Correspondence

**Neil M. Ferguson**

MRC Centre for Outbreak Analysis and Modelling

Imperial College London, Faculty of Medicine

Norfolk Place

London W2 1PG

United Kingdom

neil.ferguson@imperial.ac.uk

**Ali Albarrak**

Public Health Department

Ministry of Health

Riyadh 11176

Saudi Arabia

draalbarrak@yahoo.com

## Appendix 1

### Supplemental Text: Simulation Framework

Ten thousand simulations were performed using the following procedure:

- A number of KSA resident and foreign Hajj pilgrims are drawn from Poisson distributions with rates 1.5 million and 1.7 million respectively
- The number of pilgrims in each age class is determined by assigning the *N* pilgrims to an age class based on random draws from a multinomial distribution. The age distribution of KSA pilgrims is assumed to be proportional to the distribution of KSA residents over 15 years old. The age distribution of foreign pilgrims is modeled after the distribution reported in Ebrahim 2009.
- For each age, an infection rate for the simulation, *p_ij_*is drawn from a normal distribution *Normal(p_i_, se_i_)* where *p_i_* and *se* are the mean and standard error of the age specific attack rate estimated for each scenario (i.e., the posterior of the attack rate based on the data identified for the scenario, process variation is introduced in the next step).
- The number of infections occurring during the Hajj is simulated by a random draw from a Poisson distributions: *I_ij_*~ *Poisson*(*p_ij_  * N_ij_*) , where *N_ij_* is the number of pilgrims in age class *i* in simulation *j*
- Each Case is assigned a day of infection assuming constant risk of infection over the course of the Hajj
- Up to two additional generations of transmission are simulated by seeding additional infections to subsequent days of Hajj attendance based on a random Poisson draw with rate proportional to the reproductive number (assumed to be 0.63) multiplied by the probability mass of the generation time distribution occurring on that day (as specified in Cauchemez 2014).
- Each case is assigned a day of symptoms onset based on a random draw from a log-normal distribution with median 5.6 and dispersion 1.6. Cases with a day of symptom onset of greater than 14 (7 for Saudis) are assumed to develop clinical symptoms after returning home from the Hajj
- Each case is randomly assigned a country of origin and day of infection assuming a constant risk of infection and that the distribution of cases by country matches the distribution of  Hajj attendees (Khan 2010)

## Appendix 2

### Sensitivity to over-dispersion

To account for the possibility of over-dispersion in the offspring distribution we re-ran our simulations using a highly over-dispersed negative binomial distribution (CV~100). Results are shown below:

**Table:**

	**Expected/Low ** **Incidence**		**High ** **Incidence**		**Very High ** **Incidence**	
	**Hajj pilgrims developing symptoms in KSA**	**Hajj pilgrims developing symptoms after returning**	**Hajj pilgrims developing symptoms in KSA**	**Hajj pilgrims developing symptoms after returning**	**Hajj pilgrims developing symptoms in KSA**	**Hajj pilgrims developing symptoms after returning**
***Poisson Estimates***						
**Total**	6.2(1, 17)	5.2(0, 14)	11.7(5, 20)	9.5(4, 17)	47.6(32, 66)	40.0(26, 56)
KSA Residents^*^	1.2(0, 3)	1.2(0, 4)	2.9(0, 7)	2.4(0, 7)	11.8(6, 19)	11.0(4, 20)
Visitors	5(0, 15)	4(0,12)	8.7(3, 16)	7.1(2, 14)	35.7(22, 52)	29.0(17, 43)
**Negative Binomial Estimates**						
**Total**	6.2(1, 18)	5.2(0, 18)	11.8(5, 26)	9.8(3, 31)	47.8(29, 99)	40.4(21, 108)
KSA Residents^*^	1.2(0, 3.5)	1.2(0, 4.3)	2.9(0, 7)	2.4(0, 7)	11.9(6, 19)	11.1(3, 23)
Visitors	5(0, 17)	4(0,16)	8.9(3, 23)	7.4(1, 26)	35.9(19, 87)	29.3(13, 94)
